# Items

**Published:** 1904-12

**Authors:** 


### ITEMS.

WILLIAM R. WARNER & COMPANY, of Philadelphia, pharmaceutical chemists, whose celebrated house was established in 1856, has lately been honored at the Louisiana Purchase Exposition, Saint Louis, by the highest award, the Grand Prize, for pharmaceutical preparations. This house has long been known for its integrity and the chemical and pharmaceutical perfection of its products. Its many friends will be gratified to learn of the award above mentioned.

---

PHYSICIAN'S Account Book, by J. J. Taylor, M.D., 4105 Walnut street, Philadelphia. The foregoing is the title of a useful physician's account book adapted to the pocket and ruled in a way to fit the legal requirements in case it should become necessary to prove an account in court. Since its appearance, three years ago, it has steadily gained in a popularity which it deserves.

The book contains obstetric, vaccination, and death records and cash accounts. It measures 4¼ x 6¾ inches, containing over 224 pages. Prices: bound in leather, $1.00. Also bound in manilla boards with separate leather case. Price of case and two manilla books, $2.00. Subsequent manilla books to use in the case, 60 cents each; two for $1.00; three for $1.40. Also large size for desk or office use, $4.00. Address, Dr. J. J. Taylor, author and publisher, 4105 Walnut street, Philadelphia, Pa.

---

The Perpetual Visiting and Pocket Reference Book for 1905, is the title of a memorandum book and visiting list published by the Dios Chemical Company, Saint Louis, of which the following is a table of contents: explanation of signs and how to keep visiting accounts, obstetrical memoranda, clinical emergencies, poisons and antidotes, dose table, blank leaves for weekly visiting list, memorandum, addresses of nurses, clinical, obstetrical, birth, death and vaccination records, bills rendered, cash received, articles loaned, money loaned, miscellaneous, calendar 1905, 126 pages, lapel binding, red edges. This call book will be furnished by the Dios Chemical Company, Saint Louis, on receipt of 10 cents for postage.

---

The Arlington Chemical Company has published a collection of architectural and decorative sketches, suggestive of the arrangement of offices and reception rooms, for physicians. The album booklet also contains illustrations of office interiors of several prominent physicians, among the latter being the aseptic office of Dr. D. W. Harrington, of Buffalo, originally reproduced in the Journal.

---

### President Roosevelt at the World's Fair.

The World's Fair at Saint Louis, the official designation of which is "The Louisiana Purchase Exposition," is closing in a blaze of splendor just as these words are being reduced to type. The visit of the President of the United States of America,—don't forget the word America,—accompanied by Mrs. Roosevelt, Miss Alice Roosevelt, and members of his official suite, served to enliven the latter days of the exposition. One of the features of the visit of the President, which redounds to the everlasting credit of the management of the fair, is the splendid manner in which the guests included in the presidential party were safeguarded. Ex-Governor Francis and Mayor Wells are entitled to the gratitude of the American people for the executive ability displayed with such masterful skill.

